# Vegan-vegetarian low-protein supplemented diets in pregnant CKD patients: fifteen years of experience

**DOI:** 10.1186/s12882-016-0339-y

**Published:** 2016-09-20

**Authors:** Rossella Attini, Filomena Leone, Silvia Parisi, Federica Fassio, Irene Capizzi, Valentina Loi, Loredana Colla, Maura Rossetti, Martina Gerbino, Stefania Maxia, Maria Grazia Alemanno, Fosca Minelli, Ettore Piccoli, Elisabetta Versino, Marilisa Biolcati, Paolo Avagnina, Antonello Pani, Gianfranca Cabiddu, Tullia Todros, Giorgina B. Piccoli

**Affiliations:** 1 SS Nephrology, SCDU Urology, AOU San Luigi, Orbassano, Italy; 2 SS Epidemiology, University of Torino, Torino, Italy; 3 SSD Clinical Nutrition, Department of Clinical and Biological Sciences, University of Torino, Torino, Italy; 4 SCD Nephrology, Brotzu Hospital, Cagliari, Italy; 5 SCDU Nephrology, Department Città della Salute e della Scienza, University of Torino, Torino, Italy; 6 SS Nephrology, Department of Clinical and Biological Sciences, University of Torino, Regione Gonzole 10, Orbassano, Torino 10100 Italy; 7 Nèphrologie, CH du Mans, Le Mans, France

**Keywords:** Low-protein diets, Supplemented diets, Pregnancy, CKD, Maternal-foetal outcomes, Small for gestational age baby, Preterm delivery

## Abstract

**Background:**

Pregnancy in women with advanced CKD becoming increasingly common. However, experience with low-protein diets in CKD patients in pregnancy is still limited.

Aim of this study is to review the results obtained over the last 15 years with moderately restricted low-protein diets in pregnant CKD women (combining: CKD stages 3-5, proteinuria: nephrotic at any time, or > =1 g/24 at start or referral; nephrotic in previous pregnancy). CKD patients on unrestricted diets were employed for comparison.

**Methods:**

Study period: January, 2000 to September, 2015: 36 on-diet pregnancies (31 singleton deliveries, 3 twin deliveries, 1 pregnancy termination, 1 miscarriage); 47 controls (42 singleton deliveries, 5 miscarriages). The diet is basically vegan; since occasional milk and yoghurt are allowed, we defined it vegan-vegetarian; protein intake (0.6–0.8 g/Kg/day), keto-acid supplementation, protein-unrestricted meals (1–3/week) are prescribed according to CKD stage and nutritional status. Statistical analysis was performed as implemented on SPSS.

**Results:**

Patients and controls were similar (p: ns) at baseline with regard to age (33 vs 33.5), referral week (7 vs 9), kidney function (CKD 3-5: 48.4 % vs 64.3 %); prevalence of hypertension (51.6 % vs 40.5 %) and proteinuria >3 g/24 h (16.1 % vs 12.2 %). There were more diabetic nephropathies in on-diet patients (on diet: 31.0 % vs controls 5.3 %; p 0.007 (Fisher)) while lupus nephropathies were non-significantly higher in controls (on diet: 10.3 % vs controls 23.7 %; p 0.28 (Fisher)). The incidence of preterm delivery was similar (<37 weeks: on-diet singletons 77.4 %; controls: 71.4 %). The incidence of other adverse pregnancy related outcomes was non-significantly lower in on-diet patients (early preterm delivery: on diet: 32.3 % vs controls 35.7 %; birth-weight = <1.500 g: on diet: 9.7 % vs controls 23.8 %). None of the singletons in the on-diet series died, while two perinatal deaths occurred among the controls (*p* = 0.505).

The incidence of small for gestational age (SGA <10th centile) and/or extremely preterm babies (<28th week) was significantly lower in singletons from on-diet mothers than in controls (on diet: 12.9 % vs controls: 33.3 %; p: 0.04 (Fisher)).

**Conclusion:**

Moderate protein restriction in the context of a vegan-vegetarian supplemented diet is confirmed as a safe option in the management of pregnant CKD patients.

## Background

When we prescribed a low-protein diet to the first pregnant patient with severe proteinuria and diabetic nephropathy (a case which gave us the opportunity to start a “joint venture” between Nephrology and Obstetrics), we did not foresee that fifteen years later our Unit would have followed-up a few hundred pregnancies, about 5 % of which involved subjects on a protein-restricted diet [1–5]. We also did not foresee that several large studies would have challenged the “meat eaters” in favour of Mediterranean or vegetable-based diets, thus leading to reconsider the role of protein intake in the overall population, as well as in CKD [6–14]. We were mainly worried about the patient’s increasing levels of proteinuria, and we did not know what else we could do besides keeping blood pressure under control, ordering bed rest (still a widely used procedure) and checking the baby’s growth curve [1].

On the basis of the available data on hyper-filtration in CKD and on the effect of low-protein diets in reducing the “work load” on the remnant nephrons, we chose to start her on the diet that we considered the “best” one available in our hands, i.e. a low-protein, vegan, supplemented diet [15–18].

After our patient delivered a healthy male baby, adequate for gestational age, at the 30th gestational week, we started prescribing a low-protein, vegan-vegetarian diet, with a simplified qualitative schema, to other pregnant patients with severe kidney function impairment or relevant proteinuria [1]. Our first results, involving 12 pregnancies, were promising enough to double the number of patients in a few years [2, 4]. The subsequent analysis on 22 live-born singleton deliveries showed the almost paradoxical finding of better growth in children delivered by on-diet mothers as compared to children of CKD mothers on an unrestricted diet [4].

At the time of our first experiences, 1–1.2 g of proteins /Kg day was considered the “normal” protein intake, and the intake in pregnant women was often higher, thus making our diets conflicting with the common beliefs in pregnancy. However, interest in vegan-vegetarian diets grew over the following years, and they are now considered safe in all phases of life, including pregnancy and lactation, provided that vitamins and microelements were controlled and integrated when needed [19–31].

Meanwhile, we gradually integrated the recommendation that patients should avoid both excessive weight gain; this was carried out by shifting from a purely qualitative diet prescription to the present qualitative-quantitative one [2, 4] (Appendix).

The main drawback of our previous studies was the difficulty of recruiting a homogeneous control group [2, 4]. Thus, the novelty of the present analysis, which is aimed at reviewing the results gathered over 15 years, is that the results of on-diet pregnancies are compared to a composite larger control group of pregnancies with similar clinical characteristics.

## Methods

### Definitions and control policies

CKD was defined and staged according to K-DOQI guidelines, whenever possible according to pre-conceptional data. Throughout pregnancy, GFR and proteinuria were assessed by 24-h urine collections, as specified more in detail elsewhere [5].

A newborn was defined as Small for Gestational Age (SGA) when birth weight was below the 5th or below the 10th centile, according to the birth weight references that were used [32–34]. Due to the specific interest in this point, we employed both the older Italian Parazzini charts and the newer INeS (Italian Neonatal Study) charts, and analysed the two cut-points at the 5th and 10th percentile [33, 34]. Preterm delivery, early preterm delivery and extremely preterm delivery were defined as before 37, 34 and 28 completed weeks of gestational age, respectively [32].

Hypertension was defined as per the current guidelines; the antihypertensive treatment was mainly based upon a combination of alphamethyl-dopa and nifedipine, adding doxazosine, small doses of diuretics or clonidine only when absolutely needed. Treatment was adjusted at every clinical visit with a target of 120–130/60–70 mmHg [5].

The study was performed in two Italian settings: Torino and Cagliari. These are the two Centers with the greatest experience of management of CKD in pregnancy in Italy, that keep a conjoint database (TOCOS: Torino Cagliari Observational Study [5]). For the sake of this study, the cases were recruited in Torino, the controls were selected in both settings, as further specified. In both settings of care, the frequency of nephrological and obstetric visits, of blood and urine tests and of biometric and Doppler studies of uterine and umbilical arteries are tailored to the individual patient (visits: 1 week–1 month, biometry every 2–3 weeks in case of SGA babies or at risk for foetal growth restriction; Doppler assessment two-three times weekly in case of Doppler anomalies), in keeping with the Italian best practices in pregnant CKD patients [35, 36].

### The low-protein diet

The low protein diet consisted in an adaptation of the low-protein vegan diet employed in our centre, itself a simplification of the original scheme by Barsotti and Giovannetti [17, 18].

Unlike the Barsotti and Giovannetti diets, our basic schemas are simplified: the food is chosen according to a qualitative approach (allowed-forbidden), not weighed, with a protein intake of 0.6 g/Kg/day (ideal weight), and 1–3 free meals per week. To allow the patient to follow a vegan diet without the need to use legumes and cereals in each meal, we added a supplementation of alpha-keto analogues and aminoacids (Alpha-Kappa or Ketosteril according to the availability over time): 1 pill/10 Kg of ideal body weight [37, 38].

In an empirical attempt to balance the potential advantages of low-protein diets in CKD and the habit of increasing protein intake in pregnancy, we initially adjusted the diet from 0.6 to 0.6–0.8 g/Kg/day of proteins, based on pre-conception weight, usually by increasing the protein intake from the first (0.6 g/Kg/day) to the last trimester (0.8 g/Kg/day). We also increased the amino and keto-acids supplementation from 1 pill each 10 Kg to 1 pill each 8 Kg, and in patients with low body weight, even up to 1 pill each 5 Kg in late pregnancy.

At the time of the first case, no report on these issues had been found or made available by the company; no report on safety concerns was available at that time or was found at the subsequent updates.

Since patients often missed milk and yoghurt in their diets, we allowed small quantities (100–150 mL per day) in selected cases, and changed the definition of “vegan” into “vegan-vegetarian”. On the basis of the functional status, of the proteinuria levels and of the patients’ needs and preferences, in keeping with the policy applied to non-pregnant patients, we allowed 1–3 unrestricted meals per week (without protein restriction but limited in unsaturated fats and short-chain sugars).

On the account of the lack of indications on salt restriction in pregnancy, we did not restrict salt; since salt intake cannot be controlled by the analysis of the 24 h excretion in pregnancy, due to the lack of referral standards, we limited our interventions to diet counselling in the cases with severe oedema or uncontrolled hypertension.

In addition to the biochemical tests (targeted at CKD), we progressively added iron status, B12, and 25-OH vitamin D to the routine monthly tests; vitamins and iron supplements were employed on the basis of the biochemical results. Erythropoietin was used when needed, with a haemoglobin target of 10 g/dL on account of the physiological haemodilution of pregnancy.

The most recent version of the diet is reported in the Appendix.

### Indications for the diet and selection of controls

The main indications for the low-protein vegan-vegetarian diets in pregnancy were progressively broadened from the initial subjects with CKD stages 4-5 and/or nephrotic syndrome to include pregnancy in patients already on a supplemented vegetarian diet; CKD stages 3b or 3 with a progression trend before or during pregnancy; proteinuria above 3 g/day at any time of pregnancy, or proteinuria above 1 g/day at referral or in the first trimester, previous nephrotic syndrome, increase or development of proteinuria without any sign of preeclampsia, or a combination of any of these elements.

The controls were selected according to the same criteria from the Torino and Cagliari cohort. While the nephrologists’ approach was very similar, in keeping with our well-established cooperation, the Torino and Cagliari Units differed with regard to the Obstetric policy towards caesarean sections (more frequently performed in Cagliari [5]), therefore this outcome was not considered in the present study.

### Statistical analysis

Descriptive analysis was performed as appropriate (mean and standard deviation for parametric and median and range for non-parametric data). Paired *T*-test, Chi-square test, Fisher’s test, Mid-p test, and Wilcoxon’s test were used for comparisons between patients and controls and to evaluate the differences from referral to delivery in patients and controls. Significance was set at <0.05.

Statistical evaluation was performed using SPSS vers18.0 for Windows (SPSS Chicago Ill, USA).

### Ethical issues

Systematic counselling about the diet was provided. Patients were informed that few data on the supplemented diet during pregnancy were available outside of our group, furthermore, the limits and goals of the low-protein diets were extensively discussed. The importance of timely reporting of side effects or doubts was underlined; a written schema, progressively updated, was supplied. The first version is available elsewhere [5]. The most recent update is available in the Appendix.

The study was approved by the Ethics committee of the OIRM Sant’Anna (n° pratica 335; n° protocollo 11551/c28.2 del 4/3/2011). All patients signed a dedicate informed consent.

## Results

### Baseline data

The main baseline data of the 36 patients who followed the diet for at least one month and of the 31 patients who delivered a live-born singleton baby (excluded: 3 twin deliveries, 1 pregnancy termination following the mother’s wishes, 1 spontaneous miscarriage) are reported in Table 1. Two patients in the on-diet group undertook two pregnancies.

**Table 1:** Baseline data: “On-diet”: 36 pregnancies in patients who followed a supplemented vegan diet in pregnancy (31 singleton deliveries)

Case	Age (yrs)	Pre-conceptional; Referral-week	Kidney disease	sCr mg/dL (EPI-GFR mL/min)	CKD stage	PtU (g/24 h)	Pt/Alb (g/dL)	HT	Therapy at referral	BMI
1	35	pre; ICSI	Diab neph	1.2 (59)	3	2.5	5.9/2.8	Yes	Insulin, doxazosine	23.5
2	35	pre; 8w	Diab neph	1.6 (45)	4	1.8	6.5/3.8	No	Insulin	22
3	28	7w	Sponge kidney	3.2 (19)	4	0.8	6.5/3.5	No	EPO, Vit. D	22
4	37	pre; 6w	Diab neph	1.2 (58)	3	5.9	5.8/3.2	No	Insulin	22
5	32	6w	SLE	0.7 (115)	1	2.7	6.0/3.1	Yes	Pred., ASA, omeprazole, a-MD	24
6	35	pre; 7w	Reflux	3.2 (18)	4	1.0	8.4/4.5	Yes	Vit. D, b-blocker ASA	19
7	29	9w	Diab neph	1.5 (47)	3	6.3	6.6/3.6	Yes	Insulin, nifedipine	20
8	38	17w	fibrillary GN	0.6 (116)	1	3.6	5.8/2.6	No	None	22
9	32	6w	Kidney graft	1.2 (60)	2	0.5	6.9/4.0	No	Pred., CyA, ranitidine, ASA,	24
10	20	5w	SLE	0.6 (132)	1	2.5	6.8/3.3	No	Pred.	21
11	37	7w	Kidney graft	1.5 (44)	3	0.8	7.0/3.9	Yes	Pred., CyA, VitD, nifedipine, ASA EPO, ranitidine	27
12	30	6w	IgA GN	1.3 (55)	3	0.7	6.9/4.1	No	Levothyroxine	18.9
13	28	10w	IgA GN	1 (77)	2	2	6.6/3.7	No	None	19.9
14	36	pre; 5w	Diab neph	1 (68)	2	0.6	6.4/4.1	Yes	Lansoprazole, levothyroxine, ASA, Niphedipine, insulin,	21.5
15	35	7w	Diab neph	1.2 (56)	3	0.7	7.4/4.3	No	Insulin	18.2
16	40	24w	Diab neph	0.9 (76)	2	3.1	6.7/3.3	Yes	Insulin, levothyroxine, Nifedipine	24
17	36	20w	IgA GN	1.1 (64)	2	2.4	5.6/4.1	No	None	22.3
18	36	pre; 7w	SLE	2.9 (20)	4	3.4	6.5/3.9	Yes	Pred., levothyroxine, a-MD	24.5
19	38	6w	FSGS	0.6 (116)	1	2.1	6.3/3.3	No	CyA	25
20	33	pre; 8w	Kidney graft	1.3 (52)	3	0.2	7.2/3.9	Yes	Pred, TAC	30
21	31	pre; 9w	Sponge kidney	1.6 (43.3)	3	0.3	6.5/4.0	Yes	ASA, a-MD	23.4
22	33	pre; 7w	Reflux	0.7 (110.4)	1	0.8	6.0/3.7	Yes	ASA, b-bloc	21.8
23	38	pre; 6w	Pyelonephritis	1.2 (59)	3	0.2	6.5/3.9	Yes	a-MD	19.7
24	26	5w	Single kidney, previous HUS	1 (78)	2	0.3	6.8/3.8	Yes	ASA	23.4
25	41	pre; 7w	GN	0.8 (86.6)	2	0.8	7.2/3.8	Yes	ASA	33.6
26	32	6w	IgA GN	1 (74.7)	2	0.6	6.8 /3.9	No	ASA	24.9
27	36	18w	Diab neph	0.7 (106.3)	1	0.9	7.4/4.4	Yes	ASA, a-MD, Insulin	34
28	33	27w	LLAC	0.4 (136.2)	1	1.3	6.0/3.6	Yes	a-MD	29.7
29	33	14w	Unknown	0.8 (105.6)	1	2.2	5.7/3.0	No	ASA	24.8
30	32	30w	Unknown	1.7 (39.6)	3	0.1	6.2/3.6	No	/	26.7
31	31	8 w	Diab neph	1.48 (47)	3	0.1	6.78/4.68	*No*	ASA, Insulin	19.5
32 (twin)	31	21w	Diab neph	0.5 (128)	1	5.4	5.5/2.8	No	Insulin, levothyroxine	17.9
33 (twin)	37	12w	Unknown	0.7 (112)	1	0.8	7.1/3.5	No	None	31.4
34 (twin)	39	11 w	Previous PNA	0.57 (184.1)	1	0.29	6.11/3.37	No	ASA	32.32
35 (termination)	26	18w	MGN	0.6 (126)	1	5.5	5.1/2.3	No	None	19
36 (miscarriage)	37	7w	Kidney graft	1.7 (38)	3	0.1	6.9/3.9	Yes	Pred., TAC, EPO, ASA, omeprazole, Doxazosin, b-bloc	25.1
Summary data all cases Median (min-max)	34 (20–41)	7 (5–30)	_	sCr 1.05 (0.4–3.2) GFR-EPI 66.0 (18.0–184.1)	2 (1–4)	0.8 (0.1–6.3)	Pt 6.5 (5.1–8.4) Alb 3.75 (2.3–4.68)	17/36 47.2 %	_	23.4 (17.9–34.0)
Summary data Singletons Median (min-max)	33 (20–41)	7 (5–30)	_	sCr 1.20 (0.4–3.2) GFR-EPI 60.0 (18.0–136.2)	3 (1–4)	0.9 (0.1–6.3)	Pt 6.5 (5.6–8.4) Alb 3.8 (2.6–4.68)	16/31 51.6 %	_	23.4 (18.2–34.0)

Data at referral: data observed at the first follow-up in our unit

*HT* hypertension, *SLE* systemic lupus erythematosus, *IgA GN* IgA nephropathy, *FSGS* focal segmental glomerlosclerosis, *Diab Neph* diabetic nephropathy, *BMI* body mass index, *PtU* 24 hour proteinuria, *sCr* serum creatinine, *GFR* glomerular filtration rate, *SLE* systemic lupus erythematosus. *CyA* cyclosporine A, *ASA* acetyl salicylic acid, *Pred.* prednisone, *TAC* tacrolimus, *EPO* erythropoietin, *B-Bloc* beta blocker, *a-MD* alpha methyldopa, *ICSI* intracytoplasmatic sperm injection

Table 2 reports the baseline data in the control group of 47 pregnancies homogeneously selected according in Torino and Cagliari; there were 42 singleton deliveries and 5 spontaneous miscarriages.

**Table 2:** Baseline data: “controls”: 47 pregnant patients on unrestricted diet in pregnancy (22 singleton deliveries in Cagliari, 20 in Torino)

Case	Age (yrs)	Pre-conceptional; Referral-week	Kidney disease	sCr mg/dL (EPI-GFR mL/min)	CKD stage	PtU (g/24 h)	Pt/Alb (g/dL)	HT	Therapy at referral	BMI
1	33	11w	IgA GN	0.9 (84.3)	2	1.1	6.0/2.9	No	Pred	20.2
2	34	pre; 7w	SLE	0.7 (113.4)	1	1.3	5.8/3.5	No	Steroids, AZA	19.6
3	34	pre; 6w	SLE	0.7 (113.4)	1	1.3	6.3/2.9	Yes	a-MD, Pred., CyA	20.8
4	38	pre; 22w	Unknown	0.8 (93.8)	1	1.7	Na	Yes	ASA, Nifedipine, a-MD	26
5	29	pre; 5w	IgA GN	0.9 (86.7)	2	2.1	6.3/3.5	Yes	a-MD	22.1
6	26	pre; 8w	Diab neph	0.4 (144.2)	1	2.6	5.6/3.3	No	ASA, Insulin	23.4
7	35	pre; 7w	SLE, LLAC	0.5 (129.3)	1	3.9	5.2 /2.9	No	Pred	20.2
8	19	pre; 13w	SLE	0.6 (135.6)	1	4	5.9/3	No	none	18.8
9	36	pre; 13w	FSGS	0.9 (82.5)	2	4.2	5.7/3.1	Yes	ASA, a-MD	20.3
10	41	pre; 6w	SLE	1 (71.9)	2	5.4	5.5/3.6	Yes	a-MD	18.3
11	35	pre; 20w	Diab neph	1.2 (58.7)	3	0.1	Na	Yes	a-MD, Insulin	25.6
12	39	pre; 7w	SLE	1.4 (47.4)	3	0.3	6.5/3.7	Yes	Pred., AZA	23
13	32	pre; 9w	SLE	1.4 (49.8)	3	0.8	6.7/4.5	Yes	Pred	21.6
14	35	pre; 8w	IgA GN	1.4 (50)	3	1.7	6.8/3.3	No	none	24.8
15	38	pre; 7w	Unknown	1.6 (40.6)	3	1.4	6.2/4.3	No	none	23.9
16	31	6w	IgA GN	1.6 (42.6)	3	++	7/3.3	No	none	32.5
17	36	pre; 6w	GN	1.8 (35.7)	3	0.4	7.3/ 4	No	none	22.5
18	23	pre; 13w	Unknown	1.9 (36.6)	3	2.8	6.1/ 3	No	none	30.1
19	30	na	IgA GN	1.4 (50)	3	6.2	na	Yes	a-MD	21.6
20	34	pre; 12w	SLE, LLAC	2.2 (28.4)	4	1.2	5.7/3.2	Yes	a-MD, ASA, EPO	21.4
21	28	7w	Unknown	1.6 (43.9)	3	1.6	7.4/ 4.3	No	none	23.7
22	33	pre; 12w	GN	0.5 (127.6)	1	1.1	7/4	No	none	21.8
Summary data Cagliari	34 (19–41)	8 (5–22)		sCr 1.1 (0.4–2.2) GFR-EPI 65.3 (28.4–144.2)		1.6 (0.1–6.2)	Pt 6.2 (5.2–7.4) Alb 3.3 (2.9–4.5)	10 45.5 %		21.95 (18.3–32.5)
1	39	Pre; 8 w	Interstitial	1.6 (40)	3	1.3	7.3 /3.7	Yes	Felodipine, Doxazosin, Levotiroxina, ASA	26.7
2	27	14 w	Reflux	1.5 (47)	3	0.3	6.5/3.4	No	None	18.0
3	34	20 w	Chronic PN	1.5 (45)	3	2.0	Na	No	None	23.3
4	23	13 w	IgA GN	1.3 (56)	3	0.5	6.4/3.1	Yes	a-MD, ASA	22.7
5	32	Pre; 5 w	IgA GN	1.2 (58)	3	0.3	6.1/3.4	No	Steroids, Allopurinole	19.1
6	35	Pre; 8 w	IgA GN	1.3 (54)	3	0.5	6.6/2.7	Yes	a-MP, Niphedipine	24.4
7	22	27 w	Reflux	2.9 (22)	4	0.5	7.3/3.4	No	Niphedipine	22.2
8	39	Pre; 14 w	Chronic PN	1.4 (47)	3	0.2	8.1/4.0	Yes	B-bloc, ASA	18.4
9	31	20 w	Reflux	1.3 (54)	3	0.6	7.2/3.7	Yes	B-bloc, Doxazosine, Niphedipine, Isosorbide	19.5
10	25	33 w	Reflux	1.3 (57)	3	0.8	6.0/3.1	Yes	a-MP	19.3
11	35	7; w	Interstitial	1.3 (52)	3	0.6	6.0/3.2	Yes	None	25.6
12	33	Pre; 12w	Chronic PN	1.2 (60)	3	0.1	6.4/3.2	No	Clonidine, a-MP, ASA	19.7
13	30	6 w	Kidney graft	1.2 (59)	3	0.2	7.6/3.2	No	TAC, Pred, Pantoprazole, Allopurinolo	20.3
14	32	29 w	HIV neph.	1.43 (56)	3	0.4	7.0/3.1	No	Antiretroviral therapy Omeprazole	20.0
15	36	6 w	Kidney graft	1.1 (56)	3	0.1	6.9/4.7	No	Pred, CyA, Omeprazole, ASA	24.7
16	38	Pre; 8 w	single kidney	0.8 (56)	3	0.1	6.8/4.4	No	Calcium carbonate	15.6
17	27	5 w	SLE	0.6 (193.6)	1	1.45	7.09/4.37	No	ASA, Steroids	30.4
18	37	20 w	FSGS	0.7 (81.6)	2	2.33	6.56/3.62	No	none	23.6
19	26	12 w	IR e proteinuria	1.1 (101.2)	1	2	6.45/3.49	No	ASA	32.4
20	36	16 w	PNC	0.6 (122)	1	1.03	7.55/4.10	No	Thyroxine	20.2
21 (miscarriage)	38	7 w	Chronic PN, single kidney	1.9 (31)	3	0.1	7.3/5.0	No	None	24.9)
22 (miscarriage)	37	Pre; 8 w	Single kidney	0.8 (58)	3	0.1	7.6/4.7	No	None	15.6
23 (miscarriage)	36	5 w	Kidney graft	1.3 (53)	3	0.4	Na	No	CyA, AZA	25.5
24 (miscarriage)	37	Pre; 5 w	single kidney	0.9 (55)	3	0.1	na	No	Calcium carbonate,	16.2
25 (miscarriage)	30	9 w	Diab. Neph	1.4 (50)	3	0.2	6.9/4.1	No	Insuline	22.7
Summary data Torino	34 (22–39)	9 (5–33)	_	sCr 1.3 (0.6–2.9) GFR-EPI 56.0 (22.0–193.6)	3 (1–4)	0.4 (0.1–2.33)	Pt 6.9 (6.0–8.1) Alb 3.55 (2.7–5.0)	7 28.0 %	_	22.2 (15.6–32.4)
Summary data all controls: 42 singleton	33.5 (19–41)	9 (5–33)	_	sCr 1.25 (0.4–2.9) GFR-EPI 56.0 (22.0–193.6)	3 (1–4)	1.1 (0.1–6.2)	Pt 6.5 (5.2–8.1) Alb 3.4 (2.7–4.7)	17 40.5 %	_	21.95 (15.6–32.5)
P cases vs controls (singletons)	0.443	0.154	_	sCr 0.716 GFR-EPI 0.680	0.139 Chi 2	0.585	Pt 0.952 Alb 0.073	0.479 (Chi2)	_	0.237

Data at referral: data observed at the first follow-up in our unit

*HT* hypertension, *SLE* systemic lupus erythematosus, *IgA GN* IgA nephropathy, *FSGS* focal segmental glomerlosclerosis, *Diab Neph* diabetic nephropathy, *BMI* body mass index, *PtU* 24 hour proteinuria, *sCr* serum creatinine, *GFR* glomerular filtration rate, *SLE* systemic lupus (erithematosus)elim erythematosus. *CyA* cyclosporine A, *ASA* acetyl salicylic acid, *Pred.* prednisone, *TAC* tacrolimus, *EPO* erythropoietin, *B-Bloc* beta blocker, *a-MD* alpha methyldopa

The two groups are homogeneous with regard to the main clinical parameters: age (singletons only: on diet: 33 vs controls 33.5 years); and referral week (7 vs 9 weeks). CKD stage was non significantly lower in on diet patients (CKD 3-5: 48.4 % vs 64.3 %, p: 0.26), conversely, prevalence of hypertension was non significantly higher (51.6 % vs 40.5 %, p: 0.48). Nephrotic range proteinuria (16.1 % vs 12.2 %, p 0.74) was also non significantly higher in on diet patients. The combination of hypertension and proteinuria was present in 14/36 (38.9 %) on-diet patients and in 14/47 (29.8 %) controls (*p* = 0.35). There were more diabetic nephropathies in on-diet patients (on diet: 31 % vs controls: 5.3 %; p: 0.007) while lupus nephropathies were non-significantly higher in controls (on diet: 10.3 % vs controls 23.7 %; p: 0.28 (Fisher)), presumably as a reflection of the referral pattern of the individual Nephrology Units.

### Pregnancy outcomes: kidney function and proteinuria

All of the patients on the diet followed it throughout pregnancy; no diet- or supplement- related side effects were reported and abdominal discomfort, when present, was not considered related to the diet itself. According to dietary recall, compliance was good; however, especially in the second period, in which the diet was more detailed and no more merely qualitative, some patients complained that it was very intrusive in their daily life.

An increase in serum creatinine leading to a shift towards a higher CKD stage was observed in 19.4 % on-diet and 9.5 % controls (p: 0.2 (Fisher)).

Proteinuria increased significantly in both patients and controls (new onset or doubling of proteinuria: 54.8 % of on-diet subjects and 50 % of controls; p: 0.5 (Fisher)). However, serum albumin and total proteins only moderately, and non significantly decreased at the end of pregnancy (diet group: total proteins: 6.5 g/dL at start vs 5.7 g/dL at delivery, albumin 3.75 g/dL at start vs 2.9 g/dL at delivery; control group: total proteins: 6.5 vs 6.1 g/dL, albumin 3.4 vs 3.24 g/dL) (Tables 3 and 4).

**Table 3:** Maternal data at delivery: “on-diet”: 31 singleton deliveries and 3 twin deliveries

Case	sCr mg/dL (EPI-GFR mL/min)	Stage CKD	PtU g/24 h	Pt/Alb (g/dL)	Weight gain	Hospitalization	sCr mg/dL (EPI-GFR mL/min) 3 months	PtU g/die Serum Alb g/dl 3 months
1	1.8 (36)	3	6.2	4.8/1.9	9 (13.4 %)	95	2.0 (45)	3/2.5
2	1.8 (36)	3	5.6	5.7/2.8	11 (20 %)	73	1.9 (40)	4/3
3	3.7 (16)	4	2.6	6.3/3.6	9 (16 %)	55	4.5	0.3/3.6
4	2 (31)	3	1.9	5.6/2.9	9 (18 %)	47	2.1 (21)	1.5/3.1
5	0.7 (115)	1	3.4	4.8/2.9	14 (21.5 %)	123	-	-
6	2.9 (20)	4	2.0	6.2/2.9	10 (21.7 %)	80	2.8 (25)	1.5/3.5
7	5 (11)	5	17.3	4.2/1.8	16 (25 %)	93	4.3 (19)	5/3.1
8	0.6 (116)	1	2.1	5.0/2.4	10 (20 %)	30	0.8 (120)	4/3.2
9	1.3 (54)	3	3.6	5.3/2.8	8 (12 %)	84	1.2 (64)	1.3/-
10	0.5 (140)	1	2.9	5.4/2.7	11 (17 %)	63	0.7 (125.1)	6.2/2.8
11	1.8 (35)	3	5.4	5.4/2.8	5 (7 %)	99	1.7 (52.9)	6.8/3.8
12	0.8 (99)	1	5.7	5.5/2.7	10 (17.9 %)	9	1.2 (60.6)	5.7/3.2
13	1.5 (45)	3	5.5	5.0/2.6	4 (7.8 %)	28	2.9 (23.3)	3.4/4
14	1 (73)	2	4.7	4.5/2.2	12 (21 %)	29	0.9 (82.5)	4.4/2.2
15	1.5 (44)	3	9.4	5.5/2.6	8 (14 %)	24	1.9 (33.7)	1.3/5.7
16	1 (72)	2	4.4	6.4/2.8	14 (30.4 %%)	24	0.9 (80.2)	1.4/3.5
17	1.1 (65)	2	2.2	6.0/2.9	11 (20.4 %)	9	na	na
18	3.6 (15)	4	3.4	5.8/3.2	12 (17.9 %)	26	3.1 (18.5)	1.3/3.2
19	0.6 (115)	1	1.4	5.2/2.6	10 (14.1 %)	16	0.7 (110.3)	2.5/2.7
20	1.8 (37)	3	0.8	6.7/3.3	7 (9.2 %)	12	1.7 (39.1)	1/3.9
21	1.2 (60)	2	1.7	5.9/3.1	15 (23.8 %)	8	1.8 (37.2)	0.3/4.1
22	0.7 (105)	1	0.9	5.7/2.9	10 (20.4 %)	3	0.8 (98.7)	0.8/4.5
23	1 (69)	2	0.3	6.8/3.6	12 (22.6 %)	4	1.3 (53.7)	0.1/3.7
24	1.2 (63.2)	2	0.6	6.7/3.6	6 (10 %)	13	1.1 (69.5)	0.1/4
25	0.8 (90.5)	1	1.8	5.5/3.2	6 (7 %)	7	0.9 (76.6)	0.7/4.3
26	1 (75.6)	2	0.8	6.6/3.1	22 (31 %)	5	1.0 (74.7)	0.8/3.3
27	0.9 (86)	2	0.9	5.9/3.1	1 (1 %)	16	na	na
28	0.5 (131.1)	1	0.4	6.0/2.7	−2 (−2.5 %)	5	0.6 (118.9)	0.5/4.7
29	1 (89.9)	2	6.2	5.5/2.9	6 (7.9 %)	5	1.0	na
30	2 (31.6)	3	0.1	6.4/3.2	9 (15 %)	22	na	na
31	2.33 (27)	4	0.41	6.4/3.3	15 (22 %)	24	na	na
32 (twin)	0.7 (117)	1	11.8	4.1/1.8	21 (42 %)	76	0.6 (121.8)	1.5/3.7
33 (twin)	0.9 (81.4)	2	na	6.1/3.3	3 (3.3 %)	8	na	na
34 (twin)	0.6 (120)	1	0.9	5.6/3.0	24 (27.3 %)	14	na	na
Summary data (singletons)	sCr 1.2 (0.5–5.0) GFR-EPI 63.2 (11.0–140.0)	3 (1–4)	2.2 (0.1–17.3)	Pt 5.7 (4.2–6.8) Alb 2.9 (1.8–3.6)	10.0 (−2–22)	24 (3–123)	sCr 1.25 (0.6–4.5) GFR-EPI 57.15 (18.5–125.1)	PtU 1.4 (0.1–6.8) Serum Alb 3.5 (2.2–5.7)

Legend: Data at delivery: data observed at the last control before delivery (usually at hospitalization

*PtU* 24 hour proteinuria, *sCr* serum creatinine, *GFR* glomerular filtration rate, *Alb* serum albumin, *na* non available

**Table 4:** Maternal data at delivery: “controls”: 42 singleton deliveries

Case	sCr mg/dL (EPI-GFR mL/min)	Stage CKD	PtU g/24 h	Pt/Alb (g/dL)	Weight gain (Kg)	sCr mg/dL (EPI-GFR mL/min) 3 months	PtU g/die Serum Alb g/dl 3 months
1	0.9 (85)	2	1.5	5.3/2.9	10	0.9 (85)	1.7/3.7
2	0.8 (96)	1	7.2	6.9/3.3	10	0.8 (96)	0.8/4
3	0.7 (113)	1	8.8	6.3/3.1	10	0.8 (96)	0.4/3.8
4	0.8 (94)	1	1.5	na	na	na	na
5	0.9 (86)	2	1.1	5.7/2.9	15	1 (76)	1.3/ 3.7
6	0.5 (136)	1	0.8	5.9/2.9	14	0.5 (129)	0.4/4.3
7	0.5 (124)	1	4.0	5.4/2.5	14	0.5 (125)	1.4/3.2
8	0.5 (141)	1	3.7	5.7/ 3.0	10	0.6 (130)	3.1/4.3
9	1.0 (72)	2	6.2	5.1/2.8	18	1.1 (64)	3.5/ 3.7
10	1.3 (51)	3	7.9	4.7/2	12	1.3 (51)	1.3/ 3.1
11	2.3 (27)	4	8.3	na	na	na	na
12	1.4 (47)	3	2.5	6/3.3	14	1.5 (43)	2.1/na
13	1.4 (50)	3	6.3	5.2/3.1	13	1.4 (49)	1/4.1
14	1.4 (48)	3	3.6	6.4/3.0	18	1.5 (44)	3.2/na
15	1.8 (35)	3	4.4	5.6/2.8	11	1.9 (33)	5.7/3.3
16	1.6 (42)	3	1.8	6.1/3.0	8	1.3 (55)	2.4/3.9
17	1.7 (38)	3	5.6	5.9/2.9	9	1.8 (35)	5.7/3.6
18	1.7 (42)	3	5.6	6.2/3.3	7	1.9 (36)	8/4
19	1.4 (50)	3	6.2	5.3/3.3	8	0.9 (131)	1.6/4.1
20	2.0 (32)	3	5.1	5.7/3.2	4	1.8 (37)	5.4/3
21	2.3 (27.8)	4	7.1	6.8/3.1	10	2.4 (26)	2.7/4.2
22	0.5 (127)	1	0.5	7.5/3.8	17	0.7 (114)	0.8/4.6
Summary data (Cagliari)	sCr 1.35 (0.5–2.3) GFR-EPI 50.5 (27.0–141.0)	3 (1–4)	4.75 (0.5–8.8)	Pt 5.8 (4.7–7.5) Alb 3.0 (2.0–3.8)	10.5 (4–18)	sCr 1.20 (0.5–2.4) GFR-EPI 59.5 (26.0–131.0)	PtU 1.90 (0.4–8.0) Serum Alb 3.85 (3.0–4.6)
1	0.83 (121)	1	1.08	6.1/3.8	8	1.2 (57)	1.0/3.5
2	0.69 (80.5)	2	1.61	6.1/2.9	12	1.4 (66)	0.9/3.7
3	1.06 (86)	2	2.83	6.343.3	10	1.7 (39)	1.2/ns
4	0.59 (112)	1	0.1	7.01/3.58	8	1.9 (42)	2.5/3.2
5	0.83 (121)	1	1.08	6.08/3.80	8	1.2 (52)	0.8/3.7
6	0.69 (80.5)	2	1.61	6.11/2.95	10	Na	na
7	1.06 (86)	2	2.83	6.34/3.28	2	5.2 (11)	na
8	0.59 (112)	1	0.1	7.01/3.58	6	2.1 (29)	0.5/4.0
9	0.83 (121)	1	1.08	6.08/3.80	11	2.2 (29)	1.2/3.7
10	0.69 (80.5)	2	1.61	6.11/2.95	19	1.3 (57)	0.7/4.1
11	1.06 (86)	2	2.83	6.34/3.28	11	1.1 (62)	0.1/4.1
12	0.59 (112)	1	0.1	7.01/3.58	15	1.5 (45)	0.2/3.7
13	0.83 (121)	1	1.08	6.08/3.8	14	0.9 (85)	0.3/3.8
14	0.69 (80.5)	2	1.61	6.11/2.95	7	1.2 (72)	na
15	1.06 (86)	2	2.83	6.34/3.28	3	na	na
16	0.59 (112)	1	0.1	7.01/3.58	13	0.9 (55^a^)	0.1/4.2
17	0.83 (121)	1	1.08	6.08/3.80	14	na	na
18	0.69 (80.5)	2	1.61	6.11/2.95	3	na	na
19	1.06 (86)	2	2.83	6.34/3.28	2	na	na
20	0.59 (112)	1	0.1	7.01/3.58	19	na	na
Summary data Torino	sCr 0.76 (0.59–1,06) GFR-EPI 99.0 (80.5–121.0)		1.34 (0.1–2.83)	Pt 6.2 (6.08–7.01) Alb 3.44 (2.9–3.8)	10.0 (2–19)	sCr 1.35 (0.9–5.2) GFR-EPI 53.5 (11.0–85.0)	PtU 0.75 (0.1–2.5) Serum Alb 3.7 (3.2–4.2)
Summary data, all	sCr 0.83 (0.5–2.3) GFR-EPI 86.0 (27.0–141.0)		2.15 (0.1–8.8)	Pt 6.1 (4.7–7.5) Alb 3.24 (2.0–3.8)	10.0 (2–19)	sCr 1.3 (0.5–5.2) GFR-EPI 55.0 (11.0–131.0)	PtU 1.25 (0.1–8.0) Serum Alb 3.8 (3.0–4.6)
P controls vs on diet	sCr 0.018 GFR-EPI 0.018	0.390 (Chi2)	0.876	0.010	0.364	sCr 0.565 GFR-EPI 0.813	PtU 0.499 Serum Alb 0.074

Legend: Data at delivery: data observed at the last control before delivery (usually at hospitalization

*PtU* 24 hour proteinuria, *sCr* serum creatinine, *GFR* glomerular filtration rate, *Alb* serum albumin, *na* non available

^a^creatinine clearance (small size)

**Fig. 1:**
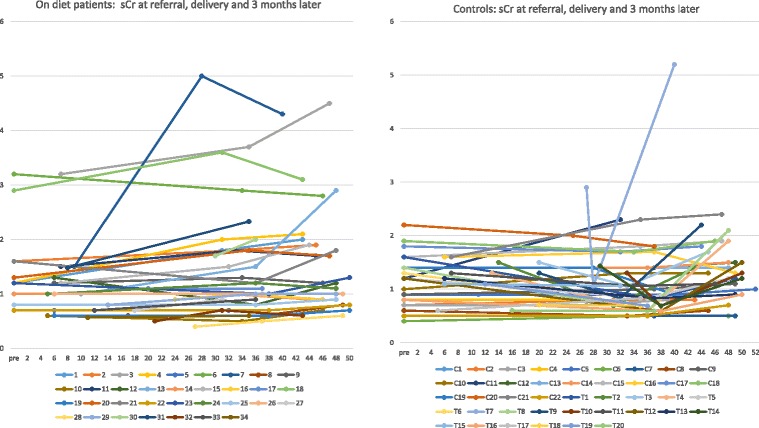
Performance of serum creatinine in on diet patients and controls

**Fig. 2:**
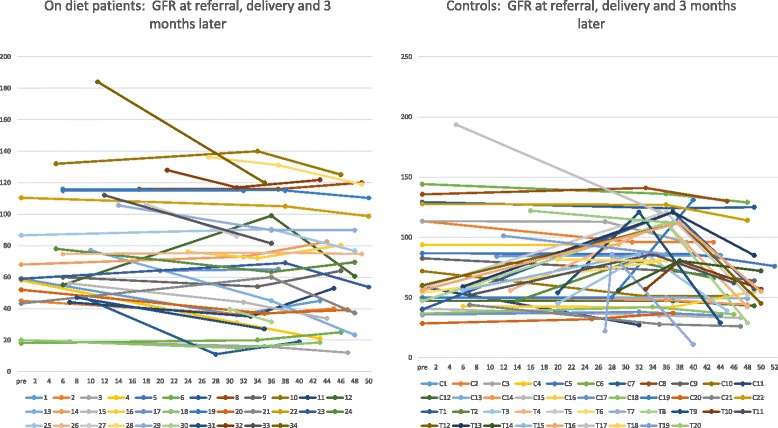
Performance of GFR in on diet patients and controls

**Fig. 3:**
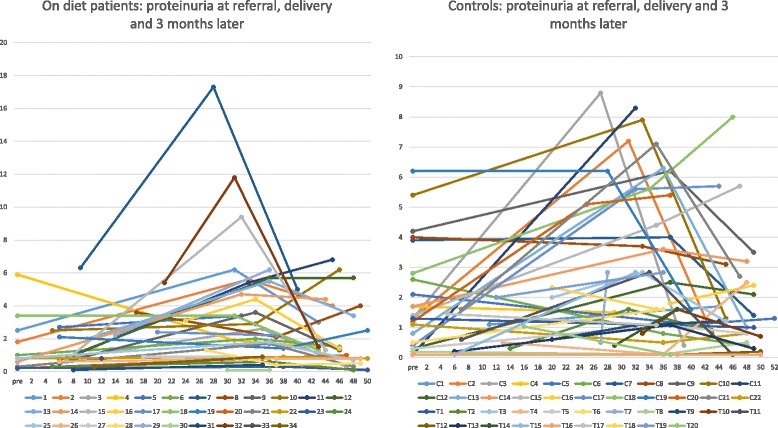
Performance of proteinuria in on diet patients and controls

At 3 months after delivery serum creatinine increased and GFR decreased in both groups, in keeping with the reversal of pregnancy-related hyperfiltration. The decrease in proteinuria is probably due both to the reversal of the hyperflitraton phase, but other less known pregnancy-related permeability changes mechanisms may also play a role (Tables 3 and 4), Figs. 1, 2 and 3.

### Pregnancy outcomes: prevalence of small for gestational age and preterm babies

Tables 5 and 6 report the main data regarding birth-weight and timing of delivery in on-diet patients and in controls. No significant differences were observed for the overall prevalence of preterm delivery (<37 completed gestational weeks), which was over 70 % in both groups (on-diet singletons 77.4 %; controls 71.4 %; p: 0.76), or in the prevalence of children with birth-weight at or below 2.5 Kg (21/31: 66.7 % vs 25/42: 59.5 %, p: 0.32).

**Table 5:** Main Maternal-foetal outcomes and intrauterine growth: “on-diet”: 31 singleton deliveries and 3 twin deliveries

Case	Gestational age Weeks (days)	Type of delivery	Sex of the baby	Weight (g)	Centile (Parazzini)	Centile (INeS)	Apgar (1–5 min)	NICU
1	31 + 0 (217)	Vaginal	M	1595	50–90	55	7–8	Yes
2	33 + 3 (234)	CS	F	1980	50–90	63	9–9	Yes
3	35 + 2 (247)	CS	F	1685	<5	5	8–9	Yes
4	31 + 0 (217)	CS	M	1970	50–90	92	8–8	Yes
5	32 + 6 (230)	CS	M	2080	50–90	75	9–9	No
6	34 + 1 (239)	CS	F	1410	<5	3	8–8	Yes
7	28 + 1 (197)	CS	F	935	10–50	42	7–8	Yes
8	37 + 1 (260)	Vaginal	M	2620	10–50	16	9–9	No
9	34 + 5 (243)	CS	M	2180	10-50	37	8–9	No
10	34 + 3 (241)	CS	F	1710	10–50	13	9–9	Yes
11	33 + 0 (231)	CS	F	2115	50–90	76	7–8	Yes
12	36 + 3 (255)	CS	F	2250	10–50	17	9–9	No
13	36 + 6 (258)	CS	F	2340	10–50	10	9–9	No
14	32 + 2 (226)	CS	F	1920	50–90	79	6–8	Yes
15	32 + 0 (224)	CS	F	1550	10–50	31	8–8	Yes
16	34 + 1 (239)	Vaginal	F	2350	50–90	93	7–8	Yes
17	37 + 4 (263)	Vaginal	F	2820	10–50	29	9–9	No
18	31 + 6 (223)	CS	M	1365	10–50	19	8–8	Yes
19	38 + 3 (269)	Vaginal	F	3180	50–90	62	9–9	No
20	35 + 5 (250)	CS	M	1790	<5	2	9–9	Yes
21	36 + 1 (253)	Vaginal	F	2140	5–10	11	9–9	No
22	38 + 6 (272)	Vaginal	F	2760	10–50	12	9–9	No
23	38 + 5 (271)	Vaginal	F	3000	10–50	29	9–9	No
24	36 + 6 (258)	Vaginal	F	2600	10–50	29	8–8	No
25	36 + 5 (257)	Vaginal	F	2740	10–50	44	9–9	No
26	37 + 2 (261)	Vaginal	M	2580	10–50	18	8–9	No
27	31 + 6 (223)	CS	F	1670	10–50	56	8–8	Yes
28	37 + 1 (260)	Vaginal	M	3070	10–50	55	9–9	No
29	36 + 6 (258)	Vaginal	F	2830	10–50	50	9–9	No
30	36 + 1 (253)	Vaginal	F	2250	10–50	22	9–9	No
31	35 + 6 (251)	CS	F	2020	10–50	23	9/9	No
32 (twin)	31 + 4 (221)	CS	^a^M	1270	5–10	16	4–7	Yes
			F	1275	10–50	22	7–8	Yes
33 (twin)	36 + 4 (256)	CS	F	2350	10–50	16	9–9	No
			M	2400	10–50	12	8–9	No
34 (twin)	35 + 6 (251)	CS	M	2920	50–90	72	8/9	No
			M	3040	50–90	81	8/9	No
Summary data: singletons	Below 37w: 24 (77.4 %) Below 34w: 10 (32.3 %) Below 28: 0 Median 35 (28–38)	CS 17 (54.8 %)	M 9 (29.0 %)	Below 1500 g: 3 (9.7 %) Below 2500 g: 21 (67.7 %) Median 2140 (935–3180)	Below 5th: 3/31 (9.7 %)	Below 5th: 2/31 (6.5 %)	5 min: 9 (6–9) 10 min 9 (8–9)	Yes 14 (45.2 %)
		Vaginal 14 (45.2 %)	F 22 (71.0 %)		Below 10th 4/31 (12.9 %)	Below 10th 3/31 (9.7 %) median 29 (2–93)		No 17 (54.8 %)

Legend: ^a^Neonatal death, *CS* caesarean section, *NICU* neonatal Intensive Care Unit, *M* male, *F* female, *Parazzini* Parazzini growth charts, *INeS* Italian Neonatal Study growth charts

**Table 6:** Main Maternal-foetal outcomes, and intrauterine growth: “controls”: 42 singleton deliveries

Case	Gestational age	Type of delivery	Sex of the baby	Weight (g)	Centile (Parazzini)	Centile (INeS)	Apgar (1–5 min)	NICU
1	32 + 5	CS	M	1470	10–50	15	6–7	Yes
2	31 + 6	CS	F	1500	10–50	41	7–9	Yes
3	27 + 3	CS	F^a^	700	/	16	7–7	Yes
4	29 + 3	CS	M	610	<5	1	4–8	Yes
5	40 + 3	CS	F	2750	10–50	7	8–10	No
6	36 + 2	CS	M	3230	50–90	86	5–7	Yes
7	37 + 1	CS	M	2340	<5	8	9–10	No
8	33 + 0	CS	F	1950	10–50	59	8–9	Yes
9	37 + 0	CS	M	2300	<5	5	9–10	No
10	33 + 5	CS	M	1900	10–50	34	9–9	Yes
11	32 + 1	CS	M	2180	50–90	93	na	Yes
12	37 + 4	CS	M	2870	10–50	27	10–10	No
13	36 + 4	CS	F	2630	10–50	37	9–10	No
14	36 + 3	CS	M	2650	10–50	32	8–9	Yes
15	35 + 4	CS	F	2400	10–50	48	10–10	No
16	37 + 1	CS	M	2970	10–50	45	8–10	No
17	32 + 0	CS	M	1950	50–90	81	8–8	Yes
18	34 + 6	CS	M	2330	10–50	49	8–10	No
19	28 + 4	CS	F	820	10–50	17	7–9	Yes
20	25 + 2	CS	M^a^	500	/	7	3–5	Yes
21	35 + 4	vaginal	F	2450	10–50	58	8–9	No
22	36 + 0	vaginal	F	2600	5–10	13	9–10	No
Summary data: Cagliari	Below 37w: 17 (77.3 %) Below 34w: 10 (45.5 %) Below 28w: 2 (9.1 %) median 34.5 (25–40)	CS 20 (90.9 %)	M 13 (59.1 %)	Below 1500 g: 5 (22.7 %) Below 2500 g: 15 (68.2 %)	Below 5th or below 28 w: 5/22 (22.7 %)	Below 5th: 1/22 (4.5 %)	5 min: 8 (3–10) 10 min 9 (5–10)	Yes 12 (54.4 %)
		Vaginal 2 (9.1 %)	F 9 (40.9 %)		Below 10th or below 28 w: 6/22 (27.3 %)	Below 10th: 5/22 (22.7 %) median 33 (1–93)		No 10 (45.5 %)
1	37 + 0	CS	F	3330	50–90	92	9–9	No
2	31 + 0	CS	M	1100	5–10	10	9–9	Yes
3	33 + 0	CS	M	1425	5–10	9	7–9	Yes
4	36 + 5	Vaginal	F	2410	10–50	24	9–9	No
5	36 + 2	Vaginal	F	2160	5–10	14	9–9	No
6	36 + 5	Vaginal	F	2600	10–50	40	9–9	No
7	28 + 2	CS	M	750	5–10	9	5–8	Yes
8	36 + 2	CS	M	2500	10–50	30	9–9	No
9	32 + 5	CS	M	1300	5–10	5	9–9	Yes
10	38 + 0	Vaginal	M	2280	<5	2	8–8	No
11	34 + 2	Vaginal	F	2160	10–50	39	8–9	No
12	38 + 3	Vaginal	F	3170	50–90	61	9–9	No
13	37 + 6	Vaginal	F	3050	50–90	59	8–8	No
14	38 + 0	CS	M	2565	5–10	6	9–9	No
15	32 + 2	CS	M	1440	10–50	19	7–9	Yes
16	38 + 4	Vaginal	M	2850	10–50	18	7–8	No
17	35 + 6	Vaginal	F	2900	50–90	85	9–9	No
18	35 + 4	CS	M	1620	<5	1	9–9	Yes
19	36 + 6	CS	F	2510	10–50	29	9–9	No
20	37 + 6	CS	M	3180	50–90	59	9–9	No
Summary data: Torino	Below 37 w: 13 (65.0 %) Below 34 w: 5 (25.0 %) Below 28 w: 0 median 36 (28–38)	CS 11 (55.0 %)	M 11 (55.0 %)	Below 1500 g: 5 (25.0 %) Below 2500 g: 10 (50.0 %) median 2455 (750–3330)	Below 5th: 2/20 (10.0 %)	Below 5th: 2/20 (10.0 %)	5 min: 8 (5–9) 10 min: 9 (8–9)	Yes 6 (30.0 %)
		Vaginal 9 (45.0 %)	F 9 (45.0 %)		Below 10th 8/20 (40.0 %)	Below 10th: 6/20 (30.0 %) median 21.5 (1–92)		No 14 (70.0 %)
Summary data: all	Below 37 w: 30 (71.4 %) Below 34 w: 15 (35.7 %) Below 28 w: 2 (4.8 %) median 35.5 (25–40)	CS 31 (73.8 %)	M 24 (57.1 %)	Below 1500 g: 10 (23.8 %) Below 2500 g: 25 (59.5 %) median 2335 (500–3330)	Below 5th: 7/42 (16.7 %) Below 10th: 14/42 (33.3 %)	Below 5th: 3/42 (7.1 %) Below 10th: 11/42 (26.2 %) median 28.0 (1–93)	5 min: 8 (5–9) 10 min: 9 (8–9)	Yes 18/42 (42.9 %)
P diet vs controls	Median: 0.839 (Mann–Whitney) Below 37: 0.759 (Chi2 Yates) Below 34: 0.954 (Chi2 Yates) Below 28: 0.505 (Fisher)	0.150 Chi2 (Yates)	0.032 Chi2 (Yates)	0.742 Mann–Whitney Below 1500 g: 0.104 (Fisher) Below 2500 g: 0.319 (Fisher)	Below 5th: 0.308 (Fisher) Below 10th: 0.040 (Fisher)	Below 5th: 0.643 (Fisher) Below 10th: 0.068 (Fisher)	5 min: 0.501 10 min: 0.076 (Mann–Whitney)	1.000 Chi2 (Yates)

Legend: ^a^Neonatal death, *CS* caesarean section, *NICU* neonatal Intensive Care Unit, *M* male, *F* female, *Parazzini* Parazzini growth charts, *INeS* Italian Neonatal Study growth charts. Fisher: one tailed test

**Fig. 4:**
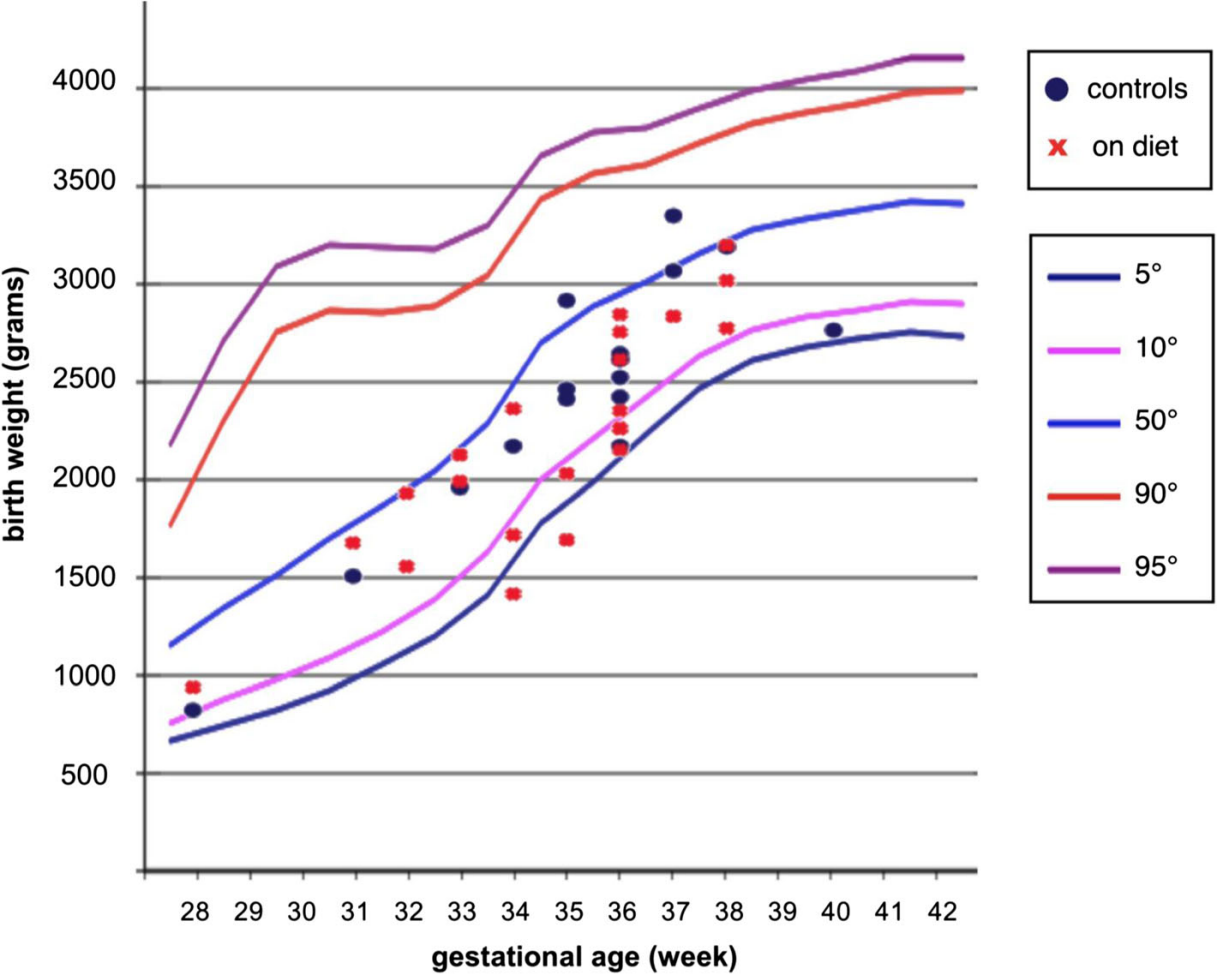
Relationship between birth-weight and prematurity in on diet patients and controls: females

**Fig. 5:**
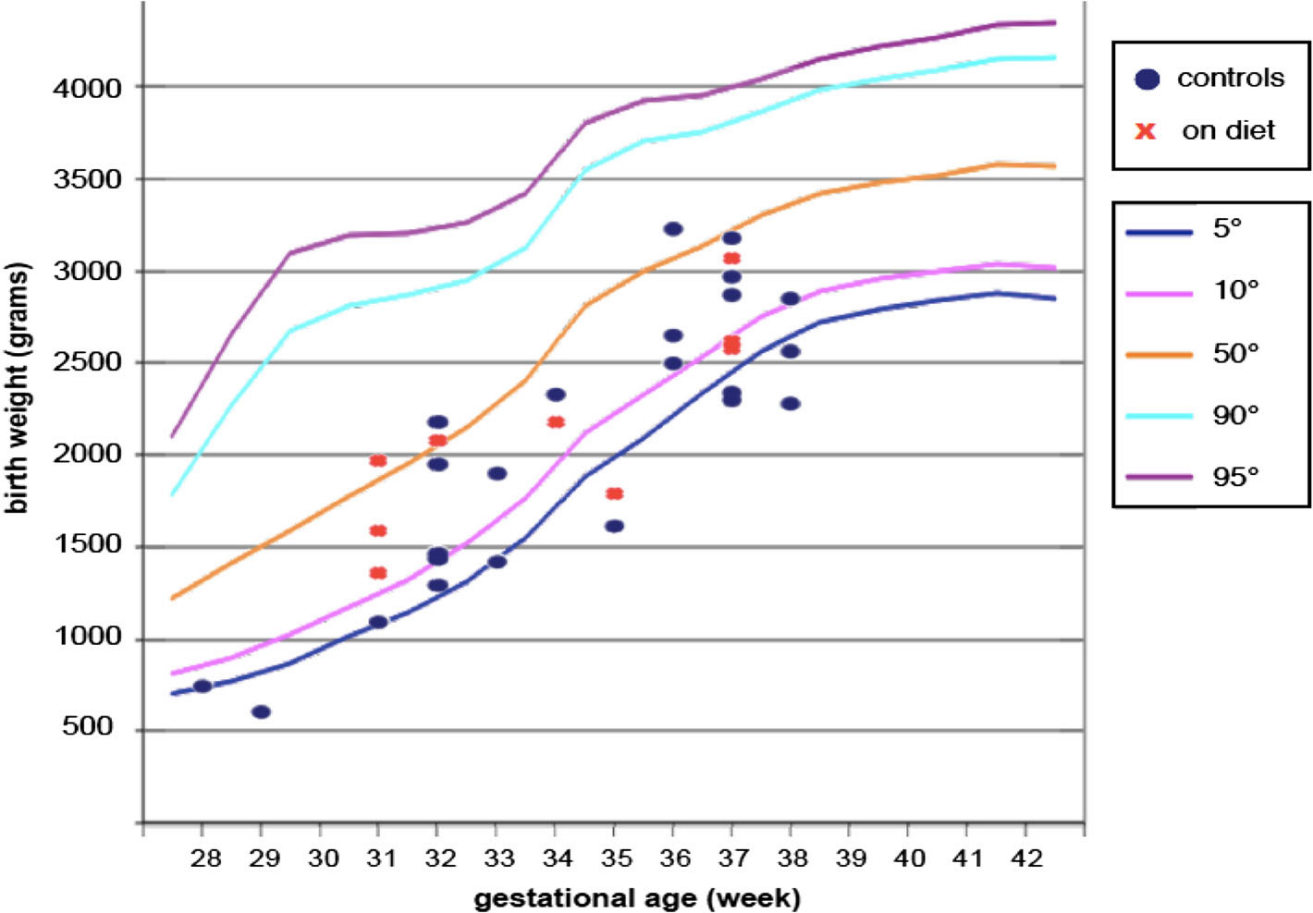
Relationship between birth-weight and prematurity in on diet patients and controls: males

The Figs. 4 and 5, based upon the original Parazzini charts that were the most commonly used references in Italy throughout the study period, summarize the relationship between birth-weight and prematurity in the two settings. Early preterm delivery (on diet: 32.3 % vs controls: 35.7 %) and extremely low birth-weight (on diet: 9.7 % vs controls: 23.8 %) were more common in control groups, and the only two extremely preterm deliveries were observed in the control group (p: 0.505).

The birth-weighty centiles, assessed by the Parazzini chart, reference in most of the period of study, showed a lower prevalence of babies below the 10th centile or extremely preterm (below 28 weeks) in on diet patients versus in controls; the difference (one tailed Fisher exact test) reaches statistical significance (12.9 % vs 33.3 % p: 0.04). If centiles are calculated with INeS charts, the figures are similar (below 10th centile: 9.7 % on diet vs 26.2 % controls, but the difference doesn’t reach statistical significance (p: 0.068)).

Conversely, gestational age and birth weight did not differ in the two cohorts (Tables 5 and 6 and Figs. 1 and 2). One twin child diet of on-diet mother died (cerebral haemorrhage after heart surgery for cardiac malformation); none of the singletons died in the on-diet series, while two perinatal deaths occurred in the control group (*p* = 0.505).

## Discussion

An often-cited quote by Feuerbach states: “a man is what he eats”; indeed there are good reasons to reflect on Feuerbach’s clever and polemic sentence in the era of epigenetics and of rediscovery of the importance of what we eat to prevent diseases and possibly to cure them.

Low protein diets are a well-known tool for contrasting absolute or relative hyperfiltration in the case of nephrotic syndrome or diabetes, and in the remnant nephrons in CKD patients [14–16, 39–41]. Pregnancy is another well-acknowledged condition of physiological hyperfiltration, which may exert a negative effect on kidney function or increase proteinuria in CKD patients [42–47].

Control of hyperfiltration and of proteinuria were the potential advantages we hoped to achieve by a low-protein diet in pregnancy, when this experience started, at a time when pregnancy in CKD was often discouraged and the common practice was to increase protein intake in pregnancy [48].

Almost unexpectedly, the finding of equivalent or better foetal growth in on-diet patients shifted our attention from the maternal kidneys to the maternal-foetal exchanges, suggesting a potential effect on the utero-placental axis [4]. While the low numbers, and the lack of a homogeneous control group limited the interest in our findings, this larger cohort with a well-matched larger control group may allow us to refine the previous results.

Similarly to our previous studies, in the present series there is a trend towards better preserved foetal growth, that reaches statistical significance for the combined outcome of extremely preterm delivery and small for gestational age baby (below the 10th centile) (Tables 5 and 6). Preterm delivery was over 70 % in cases and controls, witnessing the relevance of the renal impairment; such prevalence is in line with available studies on patients with advanced CKD [3, 5, 49–51].

In our analysis the differences between cases and controls regard the “harder” and partially overlapping outcomes, which include early preterm delivery, small for gestational age (SGA) and extreme preterm babies, “very small” babies (birth-weight is at or below1,500 g). The lower incidence of SGA has to be contextualised with the similar incidence of early preterm delivery (32.3 % vs 35.7 % in controls), since SGA is a reason for anticipating delivery [52]. This reinforces our previous findings, of a better foetal growth in children of on-diet CKD mothers (Figs. 1 and 2, Tables 5 and 6).

Our study has several limitations, which are partly shared by other studies on pregnancy: first of all, it is not randomised. However, randomization of the diet is hardly feasible outside of pregnancy and may be ethically unsound in pregnancy.

Secondly, we deal with a small number of patients, even if ours is the only study to date dedicated to this issue in CKD pregnancies.

Further research, involving a greater number of subjects is needed to highlight the differences suggested by our studies and to analyse placental vascularization and development, thus possibly offering insights into the pathogenesis of adverse pregnancy-related outcomes in CKD mothers. Theoretically, a positive effect could be due to a decrease in “vaso-toxic” elements or to an increase in “vaso-protective” ones; both are present in the study diet. A growing amount of data suggests that red meat consumption is associated with an increase in cardiovascular risk, while diets that are rich in vegetables, legumes and grains (especially those with a low glycaemic index) may be protective against endothelial dysfunction [53–63].

The specific advantage of vegetable proteins and of supplementation with ketoacids may have played an important role, as it has been suggested in experimental models, which show a protective endothelial effect of ketoacids in rats with kidney disease and a decrease in the risk of CKD in the offspring of rats with genetic kidney diseases that are fed a soya rich diet [64, 65].

In the absence of a randomised controlled trial that could present ethical limitations in pregnancy, we hope that our data may stimulate new research on this important issue.

## Conclusion

Vegan-vegetarian diets with moderate protein restriction, supplemented with amino and keto-acids, are safe in pregnancy and may be followed without appreciable side effects. A favourable trend towards improving foetal outcomes was observed for growth and timing of delivery, and reached statistical significance for the combined outcome of small for gestational age babies and extremely preterm delivery, which are also the most robust predictors of future health.

While waiting for further studies to highlight the underlying mechanisms, we hope that this positive finding may raise awareness to the important issue of diet, CKD and pregnancy.

## Appendix

### A “vegan/ vegetarian” diet for pregnant women with CKD (moderate protein restriction, supplemented with alpha-keto analogues)

**This diet is based on some very simple assumptions:**

- **first** point: a low-protein diet is associated with a reduction of the functional “work-load” for the diseased kidneys; this is important since pregnancy increases the “work-load” of the kidneys, which may increase proteinuria and thus reduce kidney function in the long term.
- **second** point: proteins of vegetable origin cause a lesser “workload” for the kidney as compared to animal-derived proteins, and therefore a “vegan-vegetarian” diet is better suited to stabilize renal function in pregnant women with chronic kidney disease.
- **third** point: vegan (no animal or animal-derived food of any kind) and vegetarian (no food from a living source: milk and derivate are allowed) diets are safe in pregnancy (regardless of the presence of CKD), if well balanced and controlled for protein deficits.
- **fourth** point: the diet should allow a good quality of life and has to be followed with flexibility, adapting to the preferences of the individual. In pregnancy, we need to pay more attention to maintaining an adequate level of several nutrients, including many vitamins and minerals (the most important ones that we know are: vitamin B12, folic acid, vitamin D, calcium), and to not gaining too much weight (this is why we ask you to pay attention not only to the quality of the food, but also to the quantity).
- **fifth** point: proteins are contained in “animal-derived” food (meat, fish, eggs, poultry and dairy products) and in plant- derived food (grains, cereals, legumes, soya, etc). While some animals (cows for example) are able to build up all the aminoacids from “energy” (i.e., grass), humans are not. Therefore, “animal-derived” proteins are called “noble” or “complete”, since they contain ALL the protein components (amino-acids) that we need; plant- derived” proteins are called “incomplete” or “non noble” since their proteins do not contain ALL the aminoacids we need. Every plant-derived protein contains some of them, but we need to put together several different types of plant- derived food to complete them.
- **sixth** point: the use of “supplements” (called alpha-kappa or ketosteril that are a mixture of essential aminoacids), allows “completion” of the vegetable proteins, avoiding the risk of nutritional deficits even in patients who do not have time (or like to) combine different “plant-derived” foods.

**Important**: There are very few studies in the medical literature on low-protein diets in pregnant CKD patients and our experience, obtained in a limited number of patients, is one of the few available ones. To date, the results have been highly positive, also thanks to the close collaboration among us (nephrologists, obstetricians, dieticians) and to the good compliance of the patients that previously followed the diet. Therefore, please report any doubts or side effects immediately to help us follow you better and better understand what we can do to improve our approach.

***In short*****:**

*You can eat anything that grows on the earth, under the earth and on trees, and anything that is derived from what grows over and under the earth and on the trees (plants). The only limit is your weight*: *be careful with fruit it is rich in sugars, and sugar is allowed “with moderation” in pregnancy (PS*: *olive oil is derived from trees, but has no sugar*: *it can be used freely).*

*You should not eat anything that walks on earth, flies in the air or swims in the water or that is derived from animals, with the exception of butter that contain mainly fat, and should be limited (not too much fat in pregnancy).*

*However, in the “free” meals you can eat ANYTHING YOU WANT (quality) but NOT TOO MUCH (quantity) to avoid gaining too much weight.*

*Supplements are prescribed as one tablet every 8–10 kg of body weight per day, sub-divided over the main meals. The tablets can be taken “in the middle of the meal”, at the beginning, or at the end of the meal. The number of pills may increase (or decrease) in pregnancy depending on your condition and biochemical test results.*

*Supplements are not required in the “free” meals.*

***In detail***

**Breakfast**

For breakfast you can have: tea, coffee, soy drink or soy yoghurt, with bread or biscuits with jam, cereal (such as corn or oat flakes or muesli), or a slice of home made cake (butter, oil, yolk, and a small quantities of milk or yoghurt are allowed) (if you cannot do without, you are allowed to have milk for breakfast…).

Alternatively, for those who prefer a savoury breakfast, you can have bread with olive oil and tomatoes or olives, bread and tofu, crackers or bread sticks with extra virgin olive oil.

1–2 (specify) ____ tablets of alpha-kappa

**Lunch and dinner (for each meal), please combine the following**

- Pasta or rice or couscous or cereals (like barley, millet, kamut, wheat) seasoned as follows (olive oil is always the best)
- Legumes (for example, chickpeas, peas, beans, lentils, etc)
- Vegetables of any kind (raw or cooked) (see indications for toxoplasmosis)
- Bread or bread sticks or crackers with extra virgin olive oil
- Fresh fruit (indicatively 150–200 g).

2–3 (specify) ____ tablets of alpha-keto analogue

Potatoes can replace bread or pasta.

Legumes should be consumed at least in one main meal in association with pasta, rice or other cereals. If you like, you can use tofu, tempeh or seitan instead of legumes.

Oily nuts such as walnuts (4–6 per day) are useful for their high content of “good” fat (that, like olive oil, protects from atherosclerosis and may help protect the placental vessels).

**Snacks**

Snacks (mid-morning, mid-afternoon) are welcome:

- 1 cup of soy yoghurt or soy drink
- Bread or biscuits or crackers or bread sticks made with extra virgin olive oil
- Bread with tofu, olives and olive paste, tomato, vegetables
- 1 cup of plain yoghurt or fruit salad
- 1 piece of fresh fruit
- raw vegetables like fennel, celery, peppers, cucumbers, tomatoes, carrots may be used if you are not receptive for toxoplasmosis (please WASH VERY WELL in any case).
- (regular yoghurt may be an alternative, at least occasionally)

**Dressings/cooking**

Cook as you prefer (stewed, steamed, grilled, broiled, baked, fried with extra virgin olive oil).

Use extra virgin olive oil for seasoning and avoid mixed seed oil, butter, lard, margarine, cream, sauces (such as mayonnaise, ketchup, tuna sauce, etc.), and items containing sodium glutamate (not good for your vessels or for the placental vessels). Also avoid: vegetable fat, non hydrogenated vegetable fats, palm oil or coconut oil (similar reasons).

Natural spices, herbs (such as rosemary, sage, basil, oregano, thyme, parsley), chili, onion, garlic, lemon juice, vinegar, balsamic vinegar, miso, tamari, shoyu, can be used; however, in case of tamari or miso, please check that they do not have added ingredients.

Use iodized salt, which should not be confused with “sea salt” or “whole salt”. Iodized salt is common salt to which iodine has been added (good for thyroid function). In order to increase the intake of some useful minerals (calcium, iron, potassium …) sesame seeds, sunflower seeds and pumpkin seeds may be added (for example 1 tablespoon).

With regard to baked goods, (crackers, bread sticks), those without added fat or made with extra virgin olive oil, sunflower or corn oil should be preferred.

**Drinks and sweets**

Drink water (still or sparkling) throughout the day. Check with your nephrologist to establish the amount. Drink wine and beer only occasionally and in small quantities during pregnancy. Cocktails, spirits and liqueurs are forbidden. Due to the high sugar content, avoid soft drinks, syrups, juices, fruit juices and soluble herbal tea.

Reduce your intake of foods with high sugar content: brown sugar, ice-cream, honey, malt, fruit jellies, croissants, cakes, cream, chocolate, cookies, candy, etc.. Avoid artificial sweeteners like aspartame (E951), acesulfame K (E950), saccharin (E954) and sucralose (E955), cyclamate (E952), neohesperidine DC (E959).

**Toxoplasmosis and listeriosis**

(Two infections that may be transmitted by food). If you are receptive to toxoplasmosis, eat only well-cooked vegetables and meat (during the free meals). CAUTION with STRAWBERRIES, berries, mushrooms, fresh herbs (like parsley, basil, sage …). In case of doubt, freeze the item to eliminate Toxoplasma. To prevent listeriosis, avoid vacuum-packed products (such as smoked salmon), raw milk, and unpasteurized cheese (such as gorgonzola, taleggio). Check the details with your dietician.

## Abbreviations

CKD: Chronic kidney disease
IUGR: Intrauterine growth restriction
PE: Preeclampsia
SGA: Small for gestational age
TOCOS: Torino Cagliari Observational Study
